# Factor Structure of the AUDIM-M Dimensional Self-Concept Questionnaire in Mexican Adolescents

**DOI:** 10.3390/children9010060

**Published:** 2022-01-04

**Authors:** José René Blanco, Martha Ornelas, Juan Cristóbal Barrón-Luján, Leticia Irene Franco-Gallegos, Susana Ivonne Aguirre, Humberto Blanco, María del Carmen Zueck, Perla Jannet Jurado-García

**Affiliations:** Faculty of Physical Culture Sciences, Autonomous University of Chihuahua, Chihuahua 31000, Mexico; jblanco@uach.mx (J.R.B.); mornelas@uach.mx (M.O.); jcbarron@uach.mx (J.C.B.-L.); lfranco@uach.mx (L.I.F.-G.); siaguirre@uach.mx (S.I.A.); hblanco@uach.mx (H.B.); mzueck@uach.mx (M.d.C.Z.)

**Keywords:** self-concept, instrumental study, factorial structure, construct validation

## Abstract

Self-concept is one of the most relevant variables in the field of personality, and a negative self-perception can pose a risk to the adolescent’s development. The present study aimed to analyze the psychometric properties proposed by Aguirre and collaborators for the dimensional self-concept questionnaire (AUDIM-M). The total sample was 560 adolescents from the city of Chihuahua, Chihuahua, with a mean age of 12.96 ± 0.88 years. The factor structure of the questionnaire was analyzed using confirmatory factor analysis. The analyses show that a four-factor structure is viable and adequate (GFI 0.964; RMSEA 0.057; CFI 0.950). The four-factor structure (personal self-concept, physical self-concept, social self-concept, and academic self-concept), according to statistical and substantive criteria, shows adequate indicators of reliability and validity adjustment. The model obtained coincides with that proposed by Aguirre et al. Improving adolescents’ self-concept undoubtedly contributes to their quality of life, hence the need for valid and reliable instruments for its measurement; this study could be a first approach for future research.

## 1. Introduction

### 1.1. Self-Concept Definition

Self-concept is one of the most relevant variables within the field of personality; from an affective and motivational perspective it is related to self-acceptance and is an indicator of psychological satisfaction and personal well-being, contributing to health and mental balance. Self-concept favors the sense of one’s own identity, establishes a frame of reference to interpret external reality and one’s own experience [1,2]. The self-concept has been widely studied in the field of psychology due to its direct participation in the individual self-regulation of present and future behavior [3,4,5,6].

Self-concept is defined in a general way as a set of attitudes with respect to the self, pointing mainly to thought, feelings and behavior. It is an organized configuration of perceptions of oneself, admissible to consciousness and knowledge [7]; the subject integrates emotions, feelings and experiences to build mental representations [8]. Self-concept plays an essential role in the complex process of psychosocial development of individuals, where its relevance is framed to understand the way in which subjects regulate their own behaviors in different contexts such as family, as well as social, academic, physical and emotional environments [9].

Shavelson, et al. [10] refer to self-concept as self-perceptions formed by experience with the environment, through environmental reinforcements and the appreciations of other people. Likewise, Cardenal and Fierro [11] defined self-concept as a set of descriptive and evaluative judgments about themselves, sustaining that the self-concept represents the way in which people represent, know and value themselves. This perception and assessment that people have of themselves determines the relationship they have with others, their psychological balance, and their performance in different areas [12,13,14]. The concept of self includes not only references to how one sees oneself, from a personal, academic, professional and social perspective, but also within the most private and personal spheres of life [15].

The physical self-concept is made up of self-perceptions of physical ability, physical condition, attractiveness and strength. The emotional or personal self-concept is explained by the dimensions of self-realization, honesty, autonomy and emotional adjustment, while the social self-concept is constituted by the basic dimensions of social competence and social responsibility [16,17,18].

Craven and Marsh [1] assert that the formation and development of self-concept is dynamic and changing [19]. Self-concept increases its multidimensionality with age: at very early ages there is no difference between the self and the environment, so there is a global, undifferentiated and specific self-concept of each situation; as age increases, a more differentiated self-concept develops progressively.

### 1.2. Self-Concept in Adolescence

Adolescence is a stage characterized by important physical, cognitive and social changes. Such changes induce adolescents to reassess their self-image in various domains that include physical appearance, academic competence and acceptance of their peers; thus, self-concept is an important determinant of personal and social adjustment during this stage [5,20]. Even though, during adolescence physical self-concept plays a central role, it is in this stage of life when the development of the general and specific self-concept is irregular, and fluctuations and differences depending on age and gender are observed [13,21,22].

It is in adolescence when the processes of identity construction, the development of new forms of thought, including the capacity for moral reasoning and the strengthening of social relationships, take place, which is why it is a key and unrepeatable stage for the consolidation of values, individual and social identity, self-esteem and personal strengths [23]. Self-concept in adolescence is defined as an indicator of personal well-being that positively influences adjusted and adaptive behaviors [21].

Thus, having a negative self-perception can pose a risk to adolescents’ development, and can lead to different problems such as: eating disorders, stress, self-harm, suicidal ideation, anxiety, lack of self-confidence, depression, family problems, tobacco addiction and alcohol, as well as insufficient school and social development [22,24,25,26,27]. A positive self-concept is valued as a desirable result in many disciplines such as education, development, sport, health, social aspects and personality [15,25,28], because it allows the development of skills, the acceptance of challenges, the ability to take risks and try new things [6], it also facilitates other important aspects of psychological well-being, including happiness and motivation [1].

Self-concept has been widely studied; it has been found that young people with a good concept of themselves are more likely to be successful, handling stressful situations well and rarely losing control. It could be said that there is probably a feedback process between self-concept and the emotional domain [29,30]. It has also been considered as a relevant aspect to obtain personal and social development [13,19]. Depressive symptoms have significant effects on general self-concept, as well as on some of the specific self-concept domains [20]. Regarding the academic context, self-concept has been related to motivation, learning, self-efficacy, performance, academic achievement, academic adaptation, success and interpersonal communication [6,14,17,28,31,32,33,34,35,36,37,38,39,40,41,42,43].

In addition, in adolescents, self-concept also influences self-perceptions such as self-esteem and satisfaction with life [44]. There is a positive correlation between physical and mental health [45] and it has also been found that adolescents who have a warm upbringing present greater self-concept [46].

Likewise, De Fraine, et al. [47] expressed that during the period of secondary education, girls and boys experience a decrease in academic self-concept, this reduction rate being faster in girls. Authors such as Amezcua and Pichardo [48] also point out that during early adolescence, self-esteem and academic achievement in women suffer a significant decrease. For Chen, et al. [49] the causal relationship of the self-concept and academic achievement for pre-adolescents seems to vary depending on the courses they take.

### 1.3. Self-Concept and Its Relationship with Other Variables

In this regard, Urquijo [6] points out that the levels of self-concept in adolescents also vary according to sex, grade and type of school they attend; significant associations between self-concept and academic performance were observed only in students from public schools, highlighting, especially in seventh year girls, that the total self-concept, academic and social is related to performance in language, while in seventh and ninth grade boys, it is related to performance in language and mathematics. By contrast, students from private schools systematically present higher self-concept and academic performance.

Furthermore, there are clear gender differences in self-concept, women have a positive perception of themselves during primary school, however, at approximately twelve years of age, they suffer a great decrease in self-confidence and acceptance of their physical image, which leads them to present a lower self-concept than men. They obtain lower scores in global, academic, physical and emotional self-concept [48,50]. Pauriyal, et al. [51] reported that the general self-concept in men was almost consistent with age, manifesting better self-concept in the physical and intellectual domains, while women had a better self-concept in the moral and social domains, and that general self-concept improved with age.

Torres, Pompa, Meza, Ancer and González [9] stated that, in the academic, social, emotional and family dimensions, women scored higher than men, while the latter presented higher scores in relation to the physical dimension. With regards to verbal academic self-concept and social responsibility, women obtain higher scores than men [52].

### 1.4. Components of Self-Concept

From the multidimensionality of the self-concept there is a clear lack of models that aim to integrate the different components or dimensions of the self-concept that can, together, provide a more complete and precise account of this construct [17]. It is easy to assess the student’s academic self-concept through the grade achieved in tests and exams; however, evaluating the self-concept that relates to feelings and perceptions of an individual is much more subjective and as such, a more difficult task [53]. Therefore, rather than overall measures, more sensitive, concrete and specific measures are proposed, that is, instruments that separately measure the areas of physical appearance, school competence, general self-concept, etc. [48].

These attempts to carefully delimit the internal structure of the self-concept, as pointed out by Fernández-Zabala, et al. [54], have been accompanied by the appearance of a series of different measurement instruments that allow assessing and identifying the different facets that make up and account for each of the domains of self-concept. Among the most used questionnaires are the self-concept form 5, the self-perception inventory, the offer self-image questionnaire, self-description questionnaires, the multidimensional self-concept scale and the self-concept questionnaire (AUDIM), which is used in the present research.

The self-concept questionnaire (AUDIM) considers four specific factors: physical self-concept, social self-concept, personal self-concept and academic self-concept. Physical self-concept refers to the particular perception of physical form, as well as the degree to which people look or feel physically strong, and skills and qualities related to the practice of physical activity and sports. The social self-concept starts from the perception of one’s own social competence when it comes to developing relationships and interacting with other people. Personal self-concept includes self-perception of the most impulsive and reactive aspects of oneself; it refers to the perception of oneself as a person who can be trusted, independent of others. The academic self-concept describes the perception that the subject has of him or herself as a student and in his or her learning performance [55].

As Fernández-Zabala, Goñi, Rodríguez-Fernández and Goñi [54] state, the self-concept instrument that is analyzed in this work (i.e., the AUDIM dimensional self-concept questionnaire), is a short questionnaire with good psychometric properties for the assessment of all the basic dimensions of self-concept. In addition, it is not limited only to differentiating the large areas of self-concept but it also includes a very wide range of particular aspects.

Despite these recognized advantages of the AUDIM questionnaire, no work has yet been carried out that confirms the cultural validity of this instrument in Mexican adolescents. Therefore, the present instrumental study [56] has been aimed at providing empirical support for the factor structure of the self-concept questionnaire (AUDIM-M), proposed by Aguirre, et al. [57], which is justified by the importance of checking the factor structure of an instrument and its psychometric equivalence in different groups. In the context of intergroup comparison, it is essential to consider the need to carry out the adaptation of a psychological measurement instrument that meets all the equivalence criteria, but above all to consider whether the same factorial structure is applicable to different groups of subjects or, more generally, to different populations [58].

## 2. Materials and Methods

### 2.1. Participants

A total of 560 adolescents participated in the study, 261 women and 299 men, all high school students from the city of Chihuahua, Chihuahua. The age of the participants ranged between 12 and 16 years, with a mean of 12.96 and a standard deviation of 0.88 years.

In order to obtain a representative sample from different schools in the City of Chihuahua, Mexico, we used convenience sampling. We defined the sample size in this way because structural equations require at least two hundred participants for the model to converge, as mentioned by Ruiz, et al. [59]. Participants in the study included secondary school students living in the city of Chihuahua, with an age range between 12 and 16 years. Only those who agreed to take part in the study and that did not have issues that prevented them from completing the questionnaire were included. Participants who did not complete the questionnaire were excluded from the study.

### 2.2. Instrument

**Figure 1:**
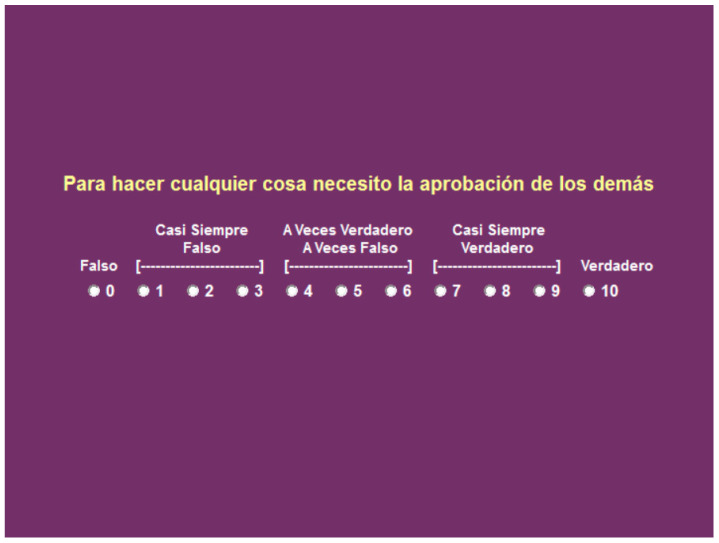
Example of response for the questionnaire items.

Dimensional self-concept questionnaire (AUDIM-M) by Aguirre, Blanco, Peinado, Mondaca and Rangel [57] is a Likert-type scale composed of 15 items related to the person (Figure 1). Participants respond on a scale from 0 to 10 (false 0, almost always false 1, 2 and 3, sometimes true, sometimes false 4, 5 and 6, almost always true 7, 8 and 9 and true 10) according to their degree of agreement with each of the proposed aspects (choosing the answer that best suits their person). The questionnaire items are grouped into four factors: personal self-concept (6 items), physical self-concept (4 items), social self-concept (3 items) and academic self-concept (2 items).

### 2.3. Procedure

In order to obtain informed consent from the participants, the educational authorities were contacted and asked to inform the directors of the selected middle schools in the city of Chihuahua about the project. Directors were requested to inform the students’ parents about the study. Once parental consent was obtained, students were invited to take part in the study. Participants completed the questionnaire on a computer; prior to accessing the instrument, they were presented with the informed assent. In order to give their assent, students were presented with two buttons, the “Yes I want to” and the “I do not want to” buttons. Students who agreed to participate had to select the “Yes I want to” button, however, if students clicked on the “I do not want to” button, the system closed the questionnaire. Students were told that they could stop completing the questionnaire at any time. The questionnaire was completed in a single 30-min session, at the beginning of which participants were given a brief introduction about the importance of the research and how to navigate the instrument. Students were required to provide honest answers and were assured that their responses would be kept confidential. Instructions on how to respond were presented on the first few screens, before the first item of the instrument. At the end of the session, students were thanked for their participation.

Once the instrument was completed, the results were collected using the results generator module of the scale editor version 2.0 (UACH, Chihuahua, MEX) [60].

The present study received approval from the Scientific Committee pertaining to the Department of Research and Postgraduate studies at the Faculty of Physical Culture Sciences of the Autonomous University of Chihuahua. In addition, the present work complies with all the regulations established in the Mexican General Health Law on Research for Health [61].

### 2.4. Data Analysis

The first step in the analysis of the psychometric properties of the questionnaire consisted of calculating the mean, standard deviations, skew, and kurtosis of each item. Items with extreme skew or kurtosis were deleted from the scale.

Two measurement models were compared: the first model (AUDIM-M4A) with four factors according to the distribution of the items proposed by Aguirre, Blanco, Peinado, Mondaca and Rangel [57] and the second model (AUDIM-M4B) that corresponds to the factor structure of the previous model, but without the items that were not sufficiently well explained by the AUDIM-M4A model.

To conduct the confirmatory factor analyses, AMOS 21 software (IBM Corp, Armonk, NY, USA) [62] was used, the variances of the error terms were specified as free parameters, for each latent variable (factor) one of the structural coefficients was set to one, so that its scale was equal to that of one of the observable variables (items). The maximum likelihood estimation method was used following the recommendation of Thompson [63], in the sense that when confirmatory factor analysis is used, not only the fit of a theoretical model should be corroborated, but it is advisable to compare the fit indices of several alternative models to select the best one.

To assess the fit of the model, the Chi-square statistic, the goodness of fit index (GFI), the standardized residual mean square root (SRMR) and the mean square error of approximation (RMSEA) were used as absolute measures of fit. The adjusted goodness of fit index (AGFI), the Tucker–Lewis index (TLI), and the comparative fit index (CFI) as measures of incremental fit. The Chi-square to degrees of freedom ratio (CMIN/GL) and the Akaike information criterion (AIC) were employed as parsimony adjustment measures [64,65].

Next, for each model, the reliability of each of the dimensions was calculated using Cronbach’s alpha coefficient [66,67] and the omega coefficient [68,69].

## 3. Results

### 3.1. Descriptive Analyses and Discrimination Indices

The descriptive analyses of each of the 15 items in the questionnaire showed that the responses to all the items reflect mean scores ranging between 4.40 and 8.32, and the standard deviation offers, in all cases, values greater than 2.00 (within a range response between 0 and 10). All skew and kurtosis values were within the range ± 2.00. Therefore, it is inferred that the variables reasonably fit a normal distribution.

### 3.2. Confirmatory Factor Analyses

The overall results of the confirmatory factor analysis (GFI 0.915; RMSEA 0.077; CFI 0.874) for the AUDIM-M4A model indicate that the measurement model is barely acceptable (Table 1).

The set of the three factors of the AUDIM-M4A model explains approximately 59% of the variance. On the other hand, 9 of the 38 items have saturations below 0.70 in their expected dimension (items 1, 2, 4, 5, 6, 8, 9, 12 and 13). Low to moderate intercorrelations between the three factors were observed showing an adequate discriminant validity between them.

The overall results of the confirmatory factor analysis (GFI 0.964; RMSEA 0.057; CFI 0.950) for the second model tested (AUDIM-M4B) that corresponds to the four-dimensional structure of the previous model without the items that were not sufficiently well explained by the AUDIM-M4A model or that according to the modification indices were not adequate (i.e., 1, 2, 6 and 12), indicate that this measurement model is better than the previous model and its adjustment is optimal (Table 1). The four factors in this model together explain approximately 66% of the variance. Furthermore, 5 of the 11 items have saturations above 0.70 in their expected dimension (items 7, 8, 11, 14 and 15). Again, low to moderate intercorrelations between the factors were observed, providing evidence for an adequate discriminant validity between them (Table 2).

### 3.3. Reliability of the Subscales (Internal Consistency)

The factors of both models present internal consistency values above 0.70 for the personal and physical self-concept factors, showing evidence of an adequate internal consistency, and below 0.70 for the social and academic self-concept factors (Table 3).

## 4. Discussion

The main goal of this study was to examine the factor structure of the self-concept questionnaire (AUDIM-M) in Mexican adolescents. The following conclusions can be drawn: The confirmatory factor analyses performed on the total sample support a four-factor structure (personal self-concept, physical self-concept, social self-concept and academic self-concept); the factors present adequate standardized factor saturations, that, in general, correspond to the structure proposed for the questionnaire by Aguirre, Blanco, Peinado, Mondaca and Rangel [57] even after deleting four of the items: two items of the personal self-concept factor (item 6: I feel happy with my body image, and item 12: I like my face), one item from the physical self-concept factor (item 1: I can run and do exercise for a long time without getting tired), and one from the social self-concept factor (item 2: I consider myself a very nervous person).

The factors, except for social self-concept, correlate with each other in a positive and statistically significant way, which shows that as the perception of self-concept improves in some of the dimensions, it also improves in the others.

The personal and physical self-concept factors showed acceptable internal consistency while the social and academic self-concept factors did not. The latter was most likely due to the reduced number of items in each factor.

Therefore, the self-concept questionnaire (AUDIM-M) is adequate to assess self-concept in adolescents. This study also serves as a premise for future research on the study of instruments for measuring self-concept in populations with different personal and cultural backgrounds.

The analysis of the psychometric properties of the AUDIM-M questionnaire has shown that a four-factor structure, according to the established psychometric requirements, is viable and adequate in adolescents. The four-factor structure has shown adequate fit and validity indicators.

## 5. Practical Applications

Some practical applications are as follows: Improving adolescents’ self-concept undoubtedly contributes to their quality of life, as has been shown in some studies where family self-esteem positively mediates the adaptation of adolescents, that is, the greater the acceptance and participation, the better they adapt, while strictness and imposition do so negatively [70], affecting even their mental health. This is due to the fact that parental rejection and overprotection reduces self-esteem and increases psychological inflexibility. By contrast, the emotional warmth of parents has a positive influence [71], hence the need to have valid and reliable instruments for its measurement. Therefore, the present study analyzes the psychometric properties proposed by Aguirre, Blanco, Peinado, Mondaca and Rangel [57] for the AUDIM-M self-concept questionnaire. This study also serves as a premise for future research on the study of instruments for measuring self-concept in populations with different personal and cultural backgrounds. Finally, this instrument will be very useful in different areas such as, for example, descriptive or intervention studies.

## 6. Limitations

At least three limitations should be mentioned for the present research. The first is that the participants were all students (limited external validity), which poses a threat to the generalizability of these results. Expanding the sample (adding, for example, adolescents who are not students) is an area of opportunity for the future. The second limitation is that no concurrent validation procedure was performed. The third limitation comes from the measurement instrument itself, which is based on self-report and therefore may contain social desirability biases. Likewise, it is essential to check whether self-concept, assessed using the AUDIM-M questionnaire, can predict healthy, academic, and social behaviors.

## Figures and Tables

**Table 1:** Absolute, incremental and parsimony indices for the generated models.

Model	Absolute Indices				Incremental Indices			Parsimony Indices	
	χ^2^	GFI	RMSEA	SRMR	AGFI	TLI	CFI	CMIN/DF	AIC
AUDIM-M4A	373.627 *	0.915	0.077	0.058	0.882	0.847	0.874	4.295	439.627
AUDIM-M4B	111.978 *	0.964	0.057	0.046	0.941	0.932	0.950	2.799	163.978

Note: * *p* < 0.05; GFI, goodness of fit index; RMSEA, root mean square error of approximation; SRMR, standardized residual mean square root; AGFI, corrected goodness of fit index; TLI, Tucker–Lewis index; CFI, comparative fit index; CMIN/DF, chi-square ratio over degrees of freedom; AIC, Akaike information criterion.

**Table 2:** Standardized solutions from the confirmatory factor analysis for the models AUDIM-M4A y AUDIM-M4B.

Item	AUDIM-M4A				AUDIM-M4B			
	F1	F2	F3	F4	F1	F2	F3	F4
**Factor Loadings**								
4 I feel happy	0.63				0.60			
6 I feel happy with my body image	0.63				-			
7 I am satisfied with the things I achieve in life	0.74				0.79			
11 I feel like a fortunate person	0.70				0.72			
12 I like my face	0.67				-			
14 I am proud of the way I am conducting my life	0.74				0.74			
1 I can run and do exercise for a long time without getting tired		0.60				-		
9 I am stronger than most people my age		0.56				0.58		
10 I have a lot of physical resistance		0.73				0.56		
15 I am physically strong		0.75				0.92		
2 I consider myself a very nervous person			0.33				-	
3 When it is time to make a decision, I greatly depend on other people’s opinion			0.78				0.42	
8 In order to do anything I need other people’s approval			0.52				0.98	
5 I am good at grammar and Spanish				0.57				0.57
13 I am good at science				0.59				0.59
**Factor Correlations**								
F1	-				-			
F2	0.45	-			0.41	-		
F3	0.00	0.00	-		0.00	0.00	-	
F4	0.52	0.42	0.00	-	0.56	0.38	0.00	-

Note: F1, personal self-concept, F2, physical self-concept, F3, social self-concept, F4, academic self-concept.

**Table 3:** Omega and alpha coefficients for the factors of the models AUDIM-M4A y AUDIM-M4B.

Factor	AUDIM-M4A		AUDIM-M4B	
	Ω	α	Ω	α
Personal self-concept	0.842	0.837	0.806	0.801
Physical self-concept	0.758	0.750	0.738	0.719
Social self-concept	0.569	0.539	0.503	0.581
Academic self-concept	0.503	0.501	0.554	0.501

## Data Availability

Data available upon request from correspondence author.
